# Associations of sleep disorders with all-cause MCI/dementia and different types of dementia – clinical evidence, potential pathomechanisms and treatment options: A narrative review

**DOI:** 10.3389/fnins.2024.1372326

**Published:** 2024-03-22

**Authors:** Geert Mayer, Helmut Frohnhofen, Martha Jokisch, Dirk M. Hermann, Janine Gronewold

**Affiliations:** ^1^Department of Neurology, Philipps-Universität Marburg, Marburg, Germany; ^2^Department of Orthopedics and Trauma Surgery, University Hospital Düsseldorf, Heinrich Heine University, Düsseldorf, Germany; ^3^Department of Medicine, Geriatrics, Faculty of Health, University Witten-Herdecke, Witten, Germany; ^4^Department of Neurology and Center for Translational Neuro-and Behavioral Sciences (C-TNBS), University Hospital Essen, University Duisburg-Essen, Essen, Germany

**Keywords:** dementia, neurocognitive disorders, Alzheimer’s disease, sleep, continuous positive airway pressure

## Abstract

Due to worldwide demographic change, the number of older persons in the population is increasing. Aging is accompanied by changes of sleep structure, deposition of beta-amyloid (Aß) and tau proteins and vascular changes and can turn into mild cognitive impairment (MCI) as well as dementia. Sleep disorders are discussed both as a risk factor for and as a consequence of MCI/dementia. Cross-sectional and longitudinal population-based as well as case–control studies revealed sleep disorders, especially sleep-disorderded breathing (SDB) and excessive or insufficient sleep durations, as risk factors for all-cause MCI/dementia. Regarding different dementia types, SDB was especially associated with vascular dementia while insomnia/insufficient sleep was related to an increased risk of Alzheimer’s disease (AD). Scarce and still inconsistent evidence suggests that therapy of sleep disorders, especially continuous positive airway pressure (CPAP) in SDB, can improve cognition in patients with sleep disorders with and without comorbid dementia and delay onset of MCI/dementia in patients with sleep disorders without previous cognitive impairment. Regarding potential pathomechanisms via which sleep disorders lead to MCI/dementia, disturbed sleep, chronic sleep deficit and SDB can impair glymphatic clearance of beta-amyloid (Aß) and tau which lead to amyloid deposition and tau aggregation resulting in changes of brain structures responsible for cognition. Orexins are discussed to modulate sleep and Aß pathology. Their diurnal fluctuation is suppressed by sleep fragmentation and the expression suppressed at the point of hippocampal atrophy, contributing to the progression of dementia. Additionally, sleep disorders can lead to an increased vascular risk profile and vascular changes such as inflammation, endothelial dysfunction and atherosclerosis which can foster neurodegenerative pathology. There is ample evidence indicating that changes of sleep structure in aging persons can lead to dementia and also evidence that therapy of sleep disorder can improve cognition. Therefore, sleep disorders should be identified and treated early.

## Graphical abstract

SDB, sleep-disordered breathing.

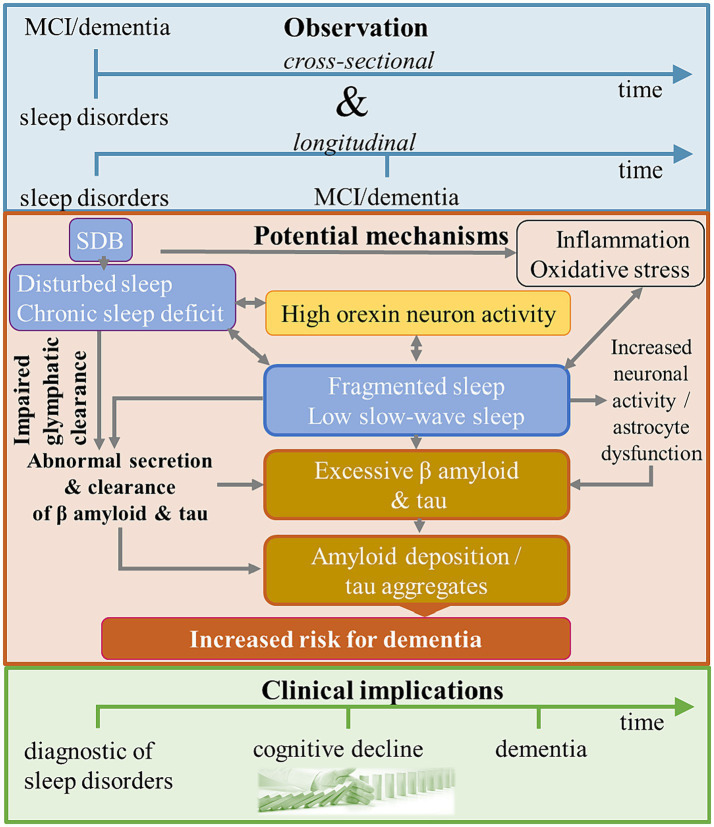

## Introduction

### Prevalence and public health relevance of dementia

Worldwide, more than 55 million people suffer from dementia with nearly 10 million new cases each year. Alzheimer’s disease (AD) is the most frequent type of dementia and contributes to about 60–70% of all dementia cases followed by vascular dementia and mixed dementia. The World Health Organization recognizes dementia as a major public health concern and developed a comprehensive blueprint for action including diagnosis, treatment, prevention and research. Concerning public health burden, dementia currently is regarded as the 7th leading cause of death and is a major cause of disability and dependency in older people. Dementia-related costs add up to more than 1 trillion US dollars, with about 50% attributable to care given by informal carers such as family members and close friends (Iadecola et al., 2019; World Health Organization, n.d.).

### Prevalence and public health relevance of sleep disorders

In the United States, the National Sleep Foundation estimates that approximately 50–70 million adults suffer from any sleep disorder, with insomnia being the most common condition with about 30% (National Sleep Foundation (NSF), n.d.). Other sleep disorders include obstructive sleep apnea (OSA), restless legs syndrome, narcolepsy, and parasomnia. OSA, characterized by repetitive pauses in breathing, affects 5–20% of adults and increases to more than 50% in older adults (Peppard et al., 2013) and more than 70% in older individuals with dementia (Frohnhofen and Roffe, 2012).

Sleep disorders not only affect sleep, but also have a profound impact on physical and mental health. Chronic untreated sleep disorders for example are associated with cardiovascular diseases such as hypertension, heart failure and stroke (Somers et al., 2008), mental disorders such as depression (Baglioni et al., 2011), reduced quality of life with impaired daytime functioning, conflicts at work and in partnership (Darchia et al., 2018) and impaired cognitive functioning, reaching from mild cognitive impairment to dementia (Bubu et al., 2017).

### The function of sleep in dementia

Sleep disorders frequently occur early in the course of dementia and affect about 44% of patients with AD and 2–2.5x more patients with vascular dementia than AD. They are highest in Lewy body dementia and Parkinson’s disease (PD, up to 90%) (Ju et al., 2013). Sleep disorders can cause multiple changes in brain structure and function (inflammation, vascular changes, degeneration, changes in glial function etc.) thus representing a risk factor for dementia or aggravating morbidity in dementia patients (Pillai and Leverenz, 2017; Iadecola et al., 2019; Gottesman et al., 2024). *Vice-versa*, changes of brain structures and functions essential for sleep regulation can cause comorbid sleep disorders in dementia patients, thus making sleep disorders and changes of brain structures and functions a bidirectional process (Mutti et al., 2021).

To understand the role of sleep for dementia it is necessary to reveal underlying neuropathologies (see Graphical abstract).

## Methods

Literature search for sleep, sleep disorders, impaired cognition, biomarkers, magnetic resonance imaging (MRI), positron emission tomography (PET), circadian rhythms, therapies and dementia, mild cognitive impairment (MCI), AD and vascular dementia was performed on Medline, Embase and Cochrane reviews. Reference lists of relevant articles were manually screened and included if they contributed to the objective of this narrative review.

### Sleep disorders as risk factor for dementia – clinical evidence

Since there is no cure for dementia yet, the prevention of dementia is highly important. Numerous risk factors for dementia are discussed, but there is still little evidence for sleep disorders compared to cardiovascular risk factors. Especially evidence regarding the association of sleep disorders and different types of dementia is insufficient (Anstey et al., 2019).

In a meta-analysis of 27 studies with a total of 69,216 participants, individuals with sleep disorders (diagnosed sleep disorders or measures of sleep quality) had a 1.68 (95% CI, 1.51–1.87) fold higher risk for the combined outcome of cognitive impairment and/or AD. For the separate outcomes of AD (clinical diagnosis), cognitive impairment (MCI based on cognitive tests), and preclinical AD (measures of AD pathology), there was a 1.55 (1.25–1.93), 1.65 (1.45–1.86), and 3.78 (2.27–6.30) fold increased risk, respectively. When the influence of different sleep disorders on the combined outcome of cognitive impairment/AD was considered, the strongest effects were seen for OSA (2.37, 1.82–3.08) and the weakest effects for insomnia (1.38, 1.13–1.67) and circadian rhythm disorders (1.38, 1.18–1.61). Based on the observation that individuals with sleep disorders had a 1.68 times higher risk for MCI and/or AD used to calculate the population-attributable risk, this meta-analysis concluded that approximately 15% of AD could be prevented by treating sleep disorders (Bubu et al., 2017).

A meta-analysis of 18 longitudinal studies including 246,786 subjects at baseline and 25,847 dementia cases investigated the association between different mostly self-reported sleep disorders and all-cause as well as different types of dementia. After an average follow-up of about 9 years, subjects who reported sleep disturbances had a 1.19 (1.11–1.29) fold higher risk of incident all-cause dementia, a 1.49 (1.25–1.78) fold higher risk of AD, and a 1.47 (1.19–1.82) fold higher risk of vascular dementia compared with subjects with normal sleep. After separate evaluation of different sleep disorders, insomnia did not increase the risk of vascular dementia (1.13, 0.94–1.35) or all-cause dementia (1.17, 0.95–1.43), but increased the risk of AD (1.51, 1.06–2.14). In contrast, SDB was associated with a higher risk of all-cause dementia (1.18, 1.02–1.36), AD (1.20, 1.03–1.41), and vascular dementia (1.23, 1.04–1.46) (Shi et al., 2018).

A meta-analysis of 11 cohort studies with a total of 1,333,424 patients focussing specifically on sleep apnea and its association with all-cause dementia and different dementia types, showed that sleep apnea patients had a 1.43 (1.26–1.62) fold higher risk of any type of incident neurocognitive disorder, AD (1.28, 1.16–1.41), PD (1.54, 1.30–1.84) and Lewy body dementia (2.06, 1.45–2.91) but not vascular dementia (1.36, 0.88–2.09) (Guay-Gagnon et al., 2022).

Longitudinal studies of population-based samples are particularly relevant for the analysis of sleep disorders as a risk factor for dementia. In larger population-based cohorts, self-reported daytime sleepiness and long sleep duration, actigraphically measured fragmented sleep and disturbed circadian rhythms were associated with an increased risk of cognitive decline, incident dementia and progression from delirium to new onset dementia (Foley et al., 2001; Benito-León et al., 2009; Tranah et al., 2011; Lim et al., 2013; Gildner et al., 2019; Gao et al., 2023). Regarding sleep duration, an inverted U-shaped association with cognitive decline was observed indicating that either insufficient or excessive sleep durations increase the risk of cognitive impairment (Brachem et al., 2020; Ma et al., 2020).

This inverted U-shaped association of sleep duration with cognitive decline was also observed after correction for APOE e4 carrier status, established AD biomarkers (cerebrospinal fluid [CSF] total tau/ Aβ42 ratio), years of education, age, and sex. Similar to sleep duration, also sleep efficiency, time in NREM, and time in REM showed an inverted U-shaped association with change in the global cognitive scores over time, meaning that high and low values were related to worsening of cognitive performance (Lucey et al., 2021). Despite previous studies suggesting that insufficient sleep/insomnia increases the risk of dementia and cognitive decline, evidence is still inconsistent. A large population-based study in older people with a long follow-up (11 years) showed no significant association of probable insomnia disorder based on diagnostic criteria with all-cause dementia or Montreal Cognitive Assessment score; similarly subjective insomnia symptoms did not increase dementia risk or a decrease in cognitive performance (Selbæk-Tungevåg et al., 2023). Similarly, a recent analysis of a large UK Biobank sample did not find a significant association of self-reported insomnia symptoms with incident all-cause or vascular dementia during a median follow-up of 13 years after adjustment for vascular risk factors; a protective effect was observed for AD (Guo et al., 2024). Another study including cognitively normal individuals as well as individuals with early and late MCI from the Alzheimer’s Disease Neuroimaging Initiative also observed that a history of insomnia (self-reported or reported by caregivers) was not significantly associated with clinical conversion to a more impaired cognitive diagnosis during a mean follow-up of 4.2 years (range 0.4–15.0 years) after adjustment for age, sex, baseline cognitive diagnosis, educational level, APOE e4 carrier status, vascular risk factors, depression, and use of sleeping pills. However, OSA history was significantly associated with a high risk of clinical conversion. Subgroup analyzes showed that OSA history increased the risk of conversion to AD in individuals with late MCI, but not in those with early MCI or cognitively normal individuals (Choe et al., 2022). In a large population-based study with a follow-up of even 40 years, any hospital-based sleep disorder diagnosis was significantly associated with increased risk of late-onset dementia only within 5 years after diagnosis suggesting an increased short-term risk of dementia following a sleep disorder diagnosis rather than evidence for an increased long-term risk. Regarding specific sleep disorders, especially sleep apnea was associated with increased dementia risk. The association between sleep disorders and dementia was stronger in men than in women and in older (≥65 years) than in younger (50–64 years) patients (Damsgaard et al., 2022). In smaller population-based studies using nocturnal polysomography (PSG), an apnea-hypopnea index (AHI) ≥15/h was associated with an increased risk for the combined outcome of MCI or dementia (Yaffe et al., 2011). Lower percentage of REM sleep and longer REM sleep latency, but not NREM sleep stages were associated with a higher risk of incident dementia (Pase et al., 2017). In addition, case–control studies confirmed that sleep apnea patients are at increased risk for incident dementia compared with age-and sex-matched controls; this is particularly true for elderly women (Chang et al., 2013).

To understand the role of sleep for dementia, it is necessary to reveal underlying neuropathologies.

### Brain structural changes in the progression to dementia and their relevance for sleep

AD – the most common type of dementia – shows several biological subtypes (Ferreira et al., 2020). It is a slowly progressing disease which begins many years prior to the manifestation of clinical symptoms and is characterized by memory loss that disrupts daily life activities. Rates of neuronal and synaptic loss indicated by the progressive rate of brain atrophy correlate with rates of cognitive decline (Dubois et al., 2016). However, global atrophy on MRI is not specific for AD. In patients who had *ante mortem* MRI the degree of atrophy correlates with *post mortem* Braak staging (Josephs et al., 2008; Whitwell et al., 2008). Braak staging is based on the spreading of neurofibrillary tangles. In Braak stages 1–2, subcortical nuclei (locus coeruleus [LC], magnocellular nuclei of the basal forebrain, hippocampal areas) are affected, in stages 3–4, neurofibrillary tangles extend up to the temporal neocortex, in stage 5 to the frontal and occipital neocortex and in stage 6 the whole brain is affected (Braak and Braak, 1991). The noradrenergic LC is a major player in the wake promoting network which together with the serotonergic raphe dorsalis projects to the basal forebrain and lateral hypothalamus (Kocsis et al., 2006). The nucleus magnocellularis has an important role in learning and memory. It is involved in the activation and desynchronisation of the cortical EEG by glutamatergic and cholinergic projections. It has a high firing rate during waking and REM sleep (Szymusiak et al., 2000). Frontobasal neurons promote sleep via descending inhibition of caudal hypothalamic and brainstem activating systems. GABAergic neurons located within magnocellular regions of the basal forebrain mediate sleep-promoting actions. Hypothalamic and brainstem afferents to the basal forebrain are functionally important for sleep wake regulation (Szymusiak, 1995). The model of sleep homeostasis is an important model for sleep wake regulation (Borbély, 2022). It is based on two processes called process S (rise in sleep pressure during waking as measured by the accumulation of slow wave sleep) and process C [circadian process controlled by the circadian clock in the nucleus suprachiasmaticus (SCN)]. Disruptions of the circadian clock leading to sleep disorders and disturbances of other circadian processes are common symptoms of aging and dementia; circadian clock disruptions may also influence processes of aging and neurodegeneration (for circadian factors other than sleep see chapter “circadian factors and the process of aging and progression to dementia”). Substantial evidence supports this bidirectional relationship between aging and deterioration of the circadian system. Age-related deterioration of circadian rhythms exacerbates multiple pathogenic processes such as altered redox homeostasis, glial dysfunction, inflammation and protein aggregation priming the brain for neurodegeneration, which may result clinically in frailty and dementia (Lananna and Musiek, 2020). Clinical evidence demonstrates that cognitively unimpaired late-middle-aged adults with increased genetic and familial risk for AD, who exibit disrupted sleep show worse cognitive performance and reduced gray matter volume including areas involved in AD (orbitofrontal and middle temporal cortex, precuneus, posterior cingulate cortex and thalamus), as well as decreased white matter diffusivity of the right hemisphere compared with those not exbibiting disrupted sleep (Grau-Rivera et al., 2020).

Although AD is the most commen type of dementia in Western countries, cognitive impairment of vascular etiology is the second most common type and may even be the predominant type in East Asia (Iadecola et al., 2019). There is still lack of consensus on the diagnostic criteria of vascular cognitive impairment. The current guidelines from the Vascular Impairment of Cognition Classification Consensus Study divides vascular cognitive impairment according to its level into mild vascular cognitive impairment and major vascular cognitive impairment (vascular dementia). Vascular dementia requires clinically significant deficits in at least 1 cognitive domain which does not have to include the memory domain which is a central symptom in AD. Often patients with vascular dementia suffer from deficits in executive function and reduced processing speed depending on the location of the vascular lesions. In contrast to AD which is a slowly progressing disease, cognition can decline gradually, stepwise or as a combition of both. In addition to a clinically relevant cognitive deficit, imaging evidence for cerebrovascular disease is required. Vascular dementia can be classified into 4 types, which are post-stroke dementia, subcortical ischemic vascular dementia, multi-infarct dementia and mixed dementia (Skrobot et al., 2018). In comparison to AD, the epidemiology and neuropathology of vascular dementia is less well understood (Iadecola et al., 2019). Often studies do not consider different dementia types. Classification into different dementia types can get difficult in older persons since mixed neurodegenerative and cerebrovascular pathology is common (Schneider et al., 2007; Kapasi et al., 2017; Power et al., 2018). Futher, evidence suggests that vascular pathology can promote neurodegenerative pathology and *vice versa* (Yarchoan et al., 2012; Sadleir et al., 2013).

### Circadian factors in the process of aging and progression to dementia

Circadian factors are changing with age. The circadian phase is advanced by up to 1 hour (Duffy et al., 2015). The phase advance is caused by a reduced exposition to natural light, body temperature and melatonin amplitude (Chellappa, 2021). The aged lens is yellowing, allowing less blue spectrum light to pass. Melanopsin retinal ganglion cells, that accumulate amyloid deposition, are reduced, therefore decreasing the input to the circadian pacemaker in the SCN. The reduced input on the SCN results in downstream changes affecting motor skills and mood, both symptoms which deteriorate in the progression of dementia (Feng et al., 2016). Age-related changes in behavioral (alcohol, smoking, nutrition etc.), socioeconomic (marital status, income etc.) and environmental (light, temperature etc.) determinants, protein clearance and orexins are other factors contributing to changes of circadian rhythm and sleep wake structure (Lee et al., 2010).

### Sleep and protein clearance in the aging process and dementia

Longitudinal investigations of solulable beta-amyloid (Aß) and tau in the interstial fluid of mice and the CSF in humans found that Aβ and tau increase during wakefulness and decrease during sleep (Barthélemy et al., 2020). There is evidence that older persons with no cognitive impairment already have Aß-plaque deposition (Mintun et al., 2006; Aizenstein et al., 2008). Aß depostion begins 20 years prior to clinical symptoms of dementia. Both Aß plaques and neurofibrillary tangles can be present in the central nervous system of non-symptomatic persons. These probably unspecific increases of Aß and tangles can be a result of the aging effects on the choroid plexus (CP), which might cause reduced clearance of the CSF from waste and toxic products (Christensen et al., 2022). The CP has a major role as a circadian clock in the central nervous system. It has its lowest CSF production at the end of the wake period and the highest in the middle of the sleep period. This rhythm feeds back to the most important circadian clock within the SCN. CSF orexins have an inverse production, highest during wakefulness and lowest during sleep. The CP fragments and metabolizes Aß, and its permeability allows Aß to penetrate into the blood, therefore contributing to a sleep-dependant clearance. The glymphatic system has a similar function as the CP. It clears the CSF from neurotoxic substances by opening the aquaporin 4 water channels at the end of glial feet only in NREM sleep. Since this clearance works during natural and anesthesized sleep, it is thought to be more dependant on sleep homeostasis than on the circadian system (Xie et al., 2013). CP is part of the glymphatic system with a regulatory function on the latter. CSF production is only reduced in AD, but not in healthy aged subjects and patients with PD (Kant et al., 2018). Despite the accumulation of Aß in the neurodegenerative process it is not increasing any more with the onset of clinical symptoms of dementia and does no longer correlate with brain atrophy, which remains correlated with tau accumulation (Josephs et al., 2008; Mormino et al., 2009).

### Sleep and amyloid

In recent years, biomarkers for dementia such as Aβ in plasma, CSF or PET have been identified predicting AD before manifestation of degenerative and cognitive symptoms (Jack Jr. et al., 2010). In older cognitively unimpaired adults, self-reported short nightly sleep duration (≤6 h) was associated with higher overall brain Aβ burden (evaluated by fluorine 18–labeled-florbetapir PET) and reduced cognitive performance mostly in memory domains compared to self-reported normal sleep duration (7–8 h). Compared to the latter, long sleep duration (≥9 h) was not significantly associated with higher Aβ burden, however it was significantly associated with worse cognitive performance in multiple domains, depressive symptoms and daytime napping (Winer et al., 2021). To investigate the age-related relationship between sleep disorders (measured by the Pittsburgh Sleep Quality Index) and AD biomarkers (CSF p-tau/Aβ42 ratio), a large group of cognitively unimpaired subjects >50 years was studied (Naismith et al., 2022). In subjects aged 50–62 years, shorter sleep duration and higher sleep efficiency were significantly associated with higher p-tau/Aβ42 ratio even when adjusted for depressive symptoms, ApOE e4 carrier status, vascular risk, hippocampal volume and WMH volume. These associations were not observed for those aged 63–69 years and for those aged 70–88 years.

Looking at region-specific brain Aβ burden [evaluated by PET with [11C] Pittsburgh Compound B (PiB)] and including cognitively unimpaired late-middle-aged adults with increased genetic and familial risk for AD, those reporting less adequate sleep, more sleep problems and greater somnolence on the Medical Outcomes Study (MOS) Sleep Scale had greater Aß burden in AD-sensitive brain regions (angular gyrus, cingulate gyrus, precuneus, and frontal medial orbital cortex). No significant correlation between Aß burden and measures of sleep duration, sleep continuity and sleep-disordered breathing (SDB) was found and brain amyloid burden was also not associated with daytime sleepiness assessed by Epworth Sleepiness Scale (ESS) (Sprecher et al., 2015). In community-dwelling cognitively unimpaired older adults without major diseases, self-reported shorter sleep duration was significantly associated with greater brain β-amyloid burden, measured by 11C-PiB PET distribution volume ratios (DVR; mean cortical DVR and precuneus DVR), whereas self-reported sleep quality was associated only with higher precuneus DVR (Spira et al., 2013).

A longitudinal study of a small sample of healthy older adults who had baseline PSG and repetitive Aß measurements by 11C-PiB PET scans showed that the proportion of NREM slow wave activity below 1 Hz (which corresponds to deep NREM sleep) and sleep efficiency predicted the speed of Aβ deposition over time (Winer et al., 2020). In another longitudinal study which included cognitively normal, MCI and AD patients, a self-reported OSA diagnosis was associated with faster annual increase in brain amyloid (increase in florbetapir PET uptake and decrease in CSF Aβ42 levels) as well as increases in CSF total tau and p-tau compared with no self-reported OSA diagnosis both in cognitively normal and MCI but not in AD patients (Bubu et al., 2019). Looking at plasma amyloid burden in cross-sectional analyzes, patients with severe OSA (AHI > 30) showed significantly higher Aβ 1–40 plasma concentration than patients with mild/moderate OSA (AHI 5–30) or controls without OSA (AHI < 5). However, Aβ 1–42 concentration did not significantly differ between the groups. Across groups, Aβ 1–40 plasma level was significantly related to hypoxia severity in PSG (Przybylska-Kuć et al., 2019). Supporting the above-mentioned observations in human studies, animal studies showed that hypoxia treatment facilitates AD pathogenesis by increasing Aβ deposition, neuritic plaque formation and memory deficits (Sun et al., 2006).

Not only chronic sleep disturbances, but also acute sleep deprivation compared to rested sleep increased brain Aβ burden in healthly controls (Shokri-Kojori et al., 2018). Comparison of multiple CSF samples during a night of unrestricted sleep vs. sleep deprivation in healthy middle-aged controls revealed that sleep deprivation interfered with a physiological morning decrease in Aβ42 in contrast to stable Aβ40, tau, and total protein levels (Ooms et al., 2014).

### The orexin system, sleep and dementia

Activating orexinergic neurons by optogenetic photostimulation increases the probability of transitions from sleep to wakefulness (Adamantidis et al., 2007). In primates, i.v. injections and nasal delivery of orexin-A reduced the negative effects of sleep deprivation on cognitive performance. This improved cognitive performance in sleep-deprived animals was associated with an increase in the activity of the dorsolateral prefrontal cortex, striatum and thalamus and an attenuation of the activation of the medial temporal lobe (Deadwyler et al., 2007). In rats, orexin-A impaired Morris water maze performance and suppressed long-term potentiation in hippocampal CA1 neurons (Aou et al., 2003). Orexin-A and orexin-B seem to have a different influence on sleep and cognition. An MRI study in mice showed that an orexin-B receptor antagonist, but not an orexin-A receptor anatagonist, increased REM, NREM and total sleep time (Gozzi et al., 2011).

*Post mortem* studies of AD patients revealed a reduction of orexin-A neurons in the hypothalamus, however CSF orexin-A concentrations were reduced compared to those of controls (Fronczek et al., 2012). Other studies found increased CSF orexin-A levels in AD and MCI patients compared with controls (Liguori et al., 2014; Gabelle et al., 2017). While there is evidence for a significant correlation of CSF orexin-A with CSF Aß42, but not CSF total tau, p-tau, cognition or subjective sleep parameters (excessive daytime sleepiness, insomnia, OSA risk, sleep duration, sleep efficiency, and wake after sleep onset) (Gabelle et al., 2017), there is also evidence for a significant correlation between CSF orexin-A levels, CSF total tau protein levels and sleep onset latency, but not between CSF Aß42 levels and cognition (Liguori et al., 2014).

### Sleep, orexin and amyloid

To examine if orexins or sleep wake patterns influence Aß, transgenic orexin-A knock-out presenilin-1 mice were investigated. The transgenic knock-out mice showed reduced Aß pathology in the brain and had an increased sleep duration. Focal overexpression of orexin in the hippocampus changed neither the total amount of sleep/wakefulness nor the Aß pathology. However, increasing wakefulness by restoring the function of orexinergic neurons in the hypothalamus increased the amount of Aß within the brain. Restoring the oxinergic neurons restored the diurnal fluctuation of Aß. Sleep depriving these animals also resulted in a significant increase of Aß plaques. The authors concluded, that modulation of sleep by orexins modulates brain Aß pathology and does not cause it (Roh et al., 2014). A recent study found that sleep fragmentation in wild type mice was associated with accumulation of Aß42 in the brain, specifically in the hippocampus, whereas it did not in orexin−/− knock-out mice (Nick et al., 2022). Orexin was able to prevent the effect of sleep fragmentation on the hippocampus, but not on LC neurons (Nick et al., 2022). This matches findings in narcoleptic patients with cataplexy and low hypocretins who had less amyloid burden in the CSF than controls (Gabelle et al., 2019). Experimental sleep fragmentation in wild type mice specifically impaired NREM sleep duration, but not REM sleep duration, which raises the question if this is another cue in the understanding of the cognitive deficits which depend largely on the reduction of slow wave sleep. Not only is orexin of key interest to the loss of LC neurons, but also sleep fragmentation itself, since sleep fragmentation led to both persistent morphological alterations in noradrenergic LC and orexinergic neurons and reductions in the cholinergic projection to the basal forebrain (Zhu et al., 2016). In contrast to animal studies, a recent meta-analysis of case–control studies assessing sleep changes by PSG in AD patients compared to healthy controls, showed that AD patients exhibited a reduced amount of REM sleep in addition to slow wave sleep and both the reduction of slow wave and REM sleep were associated with the degree of cognitive impairment as measured by Mini-Mental State Examination score. The degeneration of cholinergic regions in the brainstem and forebrain might cause slowing of EEG activity during REM sleep (Zhang et al., 2022). A study about the relationship between episodic memory and EEG frequency bands of early AD patients showed faster theta acitivity in NREM and REM sleep compared with healthy age-machted controls during postlearning sleep, which was associated with better memory performance in AD patients. These observations suggest increases in theta activation to be a compensatory mechanism to maintain cognitive performance (Hot et al., 2011).

### Sleep and the (cerebro-)vascular system

In addition to its impact on the neurogenerative process, sleep also influences vascular parameters which can contribute to neurodegeneration (Yarchoan et al., 2012; Sadleir et al., 2013). Animals studies indicated that acute sleep deprivation as a model for insomnia induces neuroinflammation (increased pro-inflammatory cytokine IL-6 levels and microglia activation in the mouse hippocampus) and impaired learning and memory in a fear conditioning paradigm (Zhu et al., 2012). In human studies, especially SDB due to intermittent hypoxia leads to vascular changes including inflammation, oxidative stress, endothelial dysfunction and atherosclerosis (Foster et al., 2007). Further, insomnia/short sleep durations and SDB increase risk factors for (cerebro-)vascular diseases including hypertension, diabetes mellitus, depression, and increased activity of the stress system (cortisol, norepinephrine, and adrenocorticotropic hormone levels, sympathetic and central activitation including whole body and brain metabolism, heart rate and pupil size) (Vgontzas et al., 2013; Gottesman et al., 2024). SDB has also shown a cross-sectional association with the presence of white matter hyperintensity which is common in vascular dementia and stroke in the middle-aged and older general population even indepent from hypertension representing a major risk factor for (cerebro-)vascular disease (Kim et al., 2013; Chokesuwattanaskul et al., 2020). Again in cross-sectional analyzes, daytime napping was significantly associated with white matter hyperintensity volume and markers of white matter ultrastructural damage in diffusion tensor imaging (Guo et al., 2024). In addition, a prospective study comprising middle-aged adults showed that self-reported short sleep duration (≤6 versus 6–8 h) predicted increased white matter hyperintensities in the parietal region 5 years later after adjustment for a variety of vascular risk factors (Yaffe et al., 2016).

### The influence of therapies of sleep disorders on the course of dementia

It is tentative to assume that in the future treatment of sleep disturbances predisposing to MCI and dementia could be effective. Unfortunately, reliable evidence from RCTs is missing so far. However, observational studies show that treatment of sleep disorders can mitigate the risk of dementia. Studies on the influence of therapy of sleep disorders on cognition in dementia patients are rare and mostly concern the influence of continuous positive airway pressure (CPAP) on cognitive outcomes in dementia patients with OSA.

To the best of the authors’ knowledge, the only double-blind placebo-controlled RCT to date, which randomized dementia patients with OSA to therapeutic CPAP therapy vs. placebo CPAP, showed significant improvement in cognition after 3 weeks of therapeutic CPAP and, after a longer follow-up of a subset of patients who continued CPAP therapy for 1 year, less cognitive deterioration, less depressive symptoms and daytime sleepiness, and better subjective sleep quality (Ancoli-Israel et al., 2008; Cooke et al., 2009).

In study designs comparing patients with OSA and PAP vs. untreated OSA, patients with OSA and PAP therapy showed no significant differences in cognitive performance cross-sectionally, but delayed onset of MCI and lower risk of dementia longitudinally (Osorio et al., 2015; Elias et al., 2018; Tsai et al., 2020; Dunietz et al., 2021).

In study designs comparing CPAP-treated OSA patients with MCI or dementia with good vs. poor CPAP adherence, those with good adherence showed better cognitive performance or less cognitive deterioration longitudinally than those with poor adherence, but no significant differences in the time of transition from MCI to dementia (Skiba et al., 2020; Wang et al., 2020; Liguori et al., 2021; Costa et al., 2023).

There is much more literature on the influence of CPAP on cognitive test performance in various domains (which, however, do not reach clinically relevant levels as in MCI or dementia) in OSA patients without previous relevant cognitive impairment/dementia. However, these studies show inconsistent results, so that the impact of CPAP on cognition in OSA patients without previous cognitive impairment is still unclear. There are major methodological problems including low numbers of participants, no or inadequate control groups or control conditions, different strategies of recruitment, severity of OSA, durations of CPAP therapy, neuropsychological test batteries and study designs. In addition, the studies do not relate cognitive changes to changes in sleep structure. Some of these problems can be addressed by meta-analyzes, which due to large sample sizes and pooling of diverse studies can analyze the influence of the aforementioned parameters (e.g., severity of OSA, duration of treatment, study design). A meta-analysis of 14 RCTs comparing therapeutic CPAP with placebo CPAP or therapy without CPAP in OSA patients showed a significant effect of CPAP on cognitive performance in the domains of attention and information processing only in patients with severe OSA (Wang et al., 2020).

A recent meta-analysis of RCTs with more heterogeneity in the study populations considered (OSA patients, insomnia patients, participants with abnormal questionnaire scores, with/without/unknown cognitive impairment) and interventions (CPAP, exercise, behavioral therapy, accupressure, magnesium supplements), with intervention durations of 12–52 weeks showed no statistically significant improvement in cognitive performance, but descriptively strong improvements in visual processing and slight improvements in long-term memory. Meta-regressions showed a significant effect of treatment duration on long-term memory with a stronger treatment effect for longer treatment durations (Franks et al., 2023).

While most studies focus on the impact of CPAP therapy on cognition/cognitive impairment/dementia in OSA patients, studies on other therapies and other sleep disorders are rare (Franks et al., 2023).

For Mandibular Advancement Splint therapy in OSA patients, crossover RCTs showed significant improvement in executive function compared to placebo tablet and no significant differences in cognitive performance compared to CPAP (Engleman et al., 2002; Barnes et al., 2004).

Uvulopalatopharyngoplasty did not change cognitive performance compared with control conditions (conservative approach such as avoidance of alcohol and weight reduction, control group with coronary bypass surgery) (Klonoff et al., 1987; Lojander et al., 1999).

Group sessions of cognitive behavioral therapy (CBT) for insomnia did not change subjective and objective cognitive performance compared with wait-list control in RCTs including patients with chronic insomnia. However, in patients with heart failure and a longer follow-up, CBT resulted in significant improvements in subjective cognition and vigilance compared with a control group which underwent an education about heart failure self-management (Perrault et al., 2022; Redeker et al., 2023). In older MCI patients with insomnia, an adapted version of CBT resulted in significant improvement of sleep and executive function, but not in memory compared with the active control nutrition class (Cassidy-Eagle et al., 2018).

Pharmacologically, insomnia can be treated by hypnotics, which are however associated with an elevated mortality rate (Kripke et al., 2012). The risk of developing dementia while on benzodiazepine intake is high (Joyce et al., 2022). Their use is approved for 1 month only and has sleep apnea as a contraindication. Since treatment of chronic sleep disorders requires long-term therapy, hypnotics with short-term application are not indicated. In a large retrospective cohort study, patients with insomnia taking hypnotics had a significantly higher risk of dementia than insomnia patients not taking hypnotics. The hypnotic-associated dementia risk was higher for insomnia patients aged 50–65 years than for insomnia patients >65 years. Dementia risk was increased for benzodiazepine and non-benzodiazepine hypnotics, short-acting, intermediate-acting, or long-acting hypnotics, low doses, medium doses, or high doses [Low: 7–30 defined daily dose (DDD) per year; medium: 31–90 DDD per year; high: ≥91 DDD per year], and previous or current but not remote use (current: prescription of hypnotics ended within 30 days before dementia index date; previous: prescription ended 31–90 days before dementia index date; remote: prescription ended ≥91 days before dementia index date). There was a non-significant tendency towards a higher dementia risk for longer half-life hypnotics compared with short-acting hypnotics. Higher doses were associated with a significantly higher dementia risk compared with lower doses. Current and previous users had a significantly higher dementia risk compared with remote hypnotic users (Chen et al., 2012).

Based on the observation that the orexin system is involved in sleep and dementia, orexin receptor antagonists are studied as a new promising treatment for insomnia with less adverse outcomes than hypnotics. In a double-blind RCT including a large number of patients with probable AD and insomnia, 4 weeks treatment with suvorexant, a dual orexin receptor antagonist, significantly increased total sleep time and sleep efficiency and decreased wake after sleep onset in PSG, but had no significant effect on the underlying sleep architecture profile and on cognition compared to placebo (Herring et al., 2020). In a small RCT with cognitively unimpaired volunteers, suvorexant treatment acutely decreased tau phosphorylation and amyloid-β in CSF by 10–20% starting 5 h after administration compared to placebo (Lucey et al., 2023).

Daridorexant, a newly approved dual orexin receptor antagonist (United States Food and Drug Administration and European Medicines Agency) could represent a future promising treatment for insomnia, since it was shown to have a favorable safety profile and high efficacy in improving sleep outcomes and also daytime functioning (Mignot et al., 2022).

Short-term application of melatonin used to modify circadian rhythm reduced sleep latency both when evaluted with PSG (Cruz-Aguilar et al., 2018, 2021) and actigraphy (Jean-Louis et al., 1998) in two placebo-controlled crossover trials with small sample sizes of patients with MCI and mild to moderate AD and comorbid sleep wake disturbances. Further, melatonin improved memory and depressed mood (Jean-Louis et al., 1998). In-depth analysis of PSG data revealed that melatonin reduced sleep latency to all stages of NREM sleep and was related to increased generation and coherence of slow EEG bands (Cruz-Aguilar et al., 2021).

Light therapy can change the phase of the circadian rhythms and has been successfully applied in senior homes to activate elderly patients. However, evidence on the efficacy of light therapy in dementia patients is still inconsistent. A recent meta-analysis about light effects (different interventions, intensities, light types) for sleep disturbances in older adults with dementia which investigated sleep measured by wrist actigraphy, sleep quality assessed by the Pittsburgh Sleep Quality Index and sleep disorders measured by the Sleep Disorders Inventory showed that night time awakenings were significantly reduced and sleep quality and circadian amplitude significantly increased. Sleep efficiency and sleep duration did not show significant changes (Tan et al., 2022). Another meta-analysis assessing sleep by actigraphy did not find significant effects on sleep (Canazei et al., 2022). In a very recent meta-analysis of RCTs evaluating the effects of light therapy on sleep, depression, neuropsychiatric behaviors, and cognition in dementia, light therapy demonstrated small-to-medium effects (Aini et al., 2023). Preliminary evidence suggests better effects when light therapy is tailored to the individual circadian phase (Cremascoli et al., 2021). A Cochrane review on non-pharmacological interventions including light therapy, social and physical activities, multimodal interventions, carer interventions, massage, and transcranial electrostimulation revealed only “very low certainty evidence” for most interventions. Only physical and social activity, carers und multimodal intervention showed “low certainty evidence” for effects on sleep parameters (Wilfling et al., 2023).

Slow oscillatory transcranial direct current stimulation (so-tDCS) could be a potential intervention to modulate sleep physiology and sleep-related memory formation. Supporting this hypothesis, so-tDCS significantly increased overall cortical slow oscillations (SO) and spindle power in frontal and centroparietal areas, amplified spindle power during SO up-phases, led to stronger synchronization between SO and spindle power fluctuations in EEG recordings and improved memory function compared with sham stimulation in patients with MCI. However, so-tDCS had no significant effect on sleep parameters (Ladenbauer et al., 2017).

Closed loop acoustic stimulation is one of the approved methods of increasing slow wave sleep and therefore should be able to increase the glymphatic clearance. It has been recently applied phase locked to slow wave sleep in 11 AD patients for the first time and showed an increase of slow wave sleep (Van den Bulcke et al., 2023).

Regarding the effect of cholinesterase inhibitors on sleep and cognition in AD patients, only donezipil led to a significant increase in REM sleep after 6 months of treatment compared to placebo in RCTs (Moraes Wdos et al., 2006; Blackman et al., 2021). During REM sleep, there was an overall reduction of theta band power and frontal delta and theta band power. REM sleep overall, and frontal and centroparietal alpha absolute power predicted cognitive improvement (Moraes Wdos et al., 2006). Interestingly, in a double-blind RCT in which patients with OSA and AD were randomized to donepezil or placebo, cognition and sleep improved significantly in the treatment group, underscoring the reciprocal relationship of sleep disturbance and cognitive impairment (Moraes et al., 2008).

## Conclusion

There is strong evidence that sleep and changes of brain structures and functions in dementia interact, which is plausible since the brain changes of normal aging subjects and demented subjects affect nuclei that are essential for the regulation of sleep and circadian rhythms. Changes of sleep structure and sleep complaints occur prior to clinical symptomatology of AD and other forms of dementia such as vascular dementia, however, a causal relationship still needs to be proven. Most studies investigating the interaction between dementia and sleep only have short follow-up times which are not sufficient to document progression. To prove the relationship, more prospective studies with long follow-ups need to be performed to show that sleep disturbances have an impact on the progression from cognitive normal to MCI and dementia. Therefore, more neurocognitive, sleep, specific biomarker data and *post mortem* brain tissue from healthy aging persons are needed. However, there is ample evidence that chronic sleep fragmentation and short sleep duration might cause changes in glymphatic Aß and tau clearance, resulting in changes in brain structures and networks important for cognition, which in the end are irreversible. Data of closed loop stimulation and use of dual orexin antagonists have shown that improvement of sleep structure, especially slow wave sleep, can improve cognitive functions in patients with MCI and reduce dementia progression. In addition, sleep disorders such as SDB and short sleep duration/insomia can lead to an increased vascular risk profile and vascular changes such as inflammation, endothelial dysfunction and atherosclerosis which can foster neurodegenerative pathology. Scarce and still inconsistent evidence suggests that therapy of sleep disorders, especially CPAP in SDB, can improve cognition in patients with sleep disorders with and without comorbid dementia and delay onset of MCI/dementia in patients with sleep disorders without previous cognitive impairment. Thus, based on the present knowledge, sleep disorders should be regarded as a predictor candidate and modifiable risk factor for dementia. Since the consequences of dementia are stressful for patients, relatives and society, early diagnosis and treatment of sleep disorders is essential in order to modify/slow disease progression. For patients not being able to tolerate or accept standard treatments, new treatment options will be needed.

## Author contributions

GM: Conceptualization, Methodology, Writing – original draft, Writing – review & editing. HF: Conceptualization, Writing – review & editing. MJ: Writing – review & editing. DH: Writing – review & editing. JG: Conceptualization, Methodology, Visualization, Writing – original draft, Writing – review & editing.
